# All Dact (Dapper/Frodo) scaffold proteins dimerize and exhibit conserved interactions with Vangl, Dvl, and serine/threonine kinases

**DOI:** 10.1186/1471-2091-12-33

**Published:** 2011-06-30

**Authors:** Saul Kivimäe, Xiao Yong Yang, Benjamin NR Cheyette

**Affiliations:** 1 The Nina Ireland Laboratory of Developmental Neurobiology, Department of Psychiatry, University of California San Francisco, 1550 4th St, San Francisco CA, 94158-2324, USA

## Abstract

**Background:**

The Dact family of scaffold proteins was discovered by virtue of binding to Dvl proteins central to Wnt and Planar Cell Polarity (PCP) signaling. Subsequently Dact proteins have been linked to a growing list of potential partners implicated in β-catenin-dependent and β-catenin-independent forms of Wnt and other signaling. To clarify conserved and non-conserved roles for this protein family, we systematically compared molecular interactions of all three murine Dact paralogs by co-immunoprecipitation of proteins recombinantly expressed in cultured human embryonic kidney cells.

**Results:**

Every Dact paralog readily formed complexes with the Vangl, Dvl, and CK1δ/ε proteins of species ranging from fruit flies to humans, as well as with PKA and PKC. Dact proteins also formed complexes with themselves and with each other; their conserved N-terminal leucine-zipper domains, which have no known binding partners, were necessary and sufficient for this interaction, suggesting that it reflects leucine-zipper-mediated homo- and hetero-dimerization. We also found weaker, though conserved, interactions of all three Dact paralogs with the catenin superfamily member p120ctn. Complex formation with other previously proposed partners including most other catenins, GSK3, LEF/TCF, HDAC1, and TGFβ receptors was paralog-specific, comparatively weak, and/or more sensitive to empirical conditions.

**Conclusions:**

Combined with published functional evidence from targeted knock-out mice, these data support a conserved role for Dact proteins in kinase-regulated biochemistry involving Vangl and Dvl. This strongly suggests that a principal role for all Dact family members is in the PCP pathway or a molecularly related signaling cascade in vertebrates.

## Background

*Dact *(Dapper antagonist of catenin; aka Dapper/Frodo) genes encode a small family of vertebrate intracellular proteins that can regulate intercellular signaling pathways [1-3]. Family members are similar in size (600-850 amino acids) and distinguished by a conserved leucine zipper motif near the N-terminus and a binding motif for PDZ (Post synaptic density-95/Discs large/Zonula occludens-1) domains at the C-terminus [1,3,4]; they also all share a few identical short (4-8 amino acid) motifs distributed elsewhere in their primary sequences [4]. The sequence surrounding the leucine zipper in some Dact family members has been suggested to be weakly homologous to Dystrophin proteins [5,6] and the region near the PDZ-binding motif is enriched for serine residues [3,6]; the functional significance of these observations is unclear. Several protein-interacting regions have been empirically delimited; these include a Lymphoid Enhancing Factor/T Cell Factor (LEF/TCF) binding region [7] a Van Gogh-like-2 (Vangl2) binding region [8], and several Dvl binding regions including the PDZ-binding motif [1,8,9]. Not so well defined are regions responsible for interactions with other proposed partners including catenins [2,10], Glycogen Synthase Kinase-3β (GSK3β) [1], 14-3-3 proteins [11], Histone Deacetylase 1 (HDAC1) [2], a subclass of TGFβ receptor proteins [12], and the zinc-finger protein DumbBell Forming-4 (DBF4) [13].

Dact1 was discovered independently by two groups conducting yeast-2-hybrid screens for partners of the Dvl scaffold protein central to the developmentally- and clinically-important Wnt signaling pathways. Initial functional analyses relied on over-expression and morpholino-based knock-down technologies in the pseudo-tetraploid frog *Xenopus laevis*. On this basis two nearly identical *Dact1 *paralogs (Dapper and Frodo) were identified and proposed to modulate both β-catenin-dependent [1,5] and β-catenin independent Wnt signaling pathways [1]. Subsequent studies in human disease and mammalian cellular models have supported a role for Dact1 in *antagonizing *Wnt/β-catenin signaling [2,14,15], whereas other studies in *Xenopus *and zebrafish have supported a role in *promoting *Wnt/β-catenin signaling [5,16]. One potential explanation for these opposing functional observations is that Wnt/β-catenin signal regulation by Dact1 could depend on phosphorylation state [11,17]. Nonetheless, a *Xenopus *Dact1 protein (Frodo) has also been shown to promote a p120-catenin (p120ctn) dependent signaling pathway that acts parallel to, but independently of, Wnt/β-catenin signaling [7,10]. Also, two independent studies using gene-targeting technology in mice have each determined that elimination of Dact1 by itself does not significantly alter Wnt/β-catenin signaling but instead causes β-catenin-independent effects on development via disruptions in the post-translational regulation of Dvl [18] and Vangl2 [8]. The notion that Dact1 primarily functions in β-catenin-independent pathways is further supported by overexpression and knock-out experiments in other developmental systems, which have demonstrated robust effects on activities of the small GTPases Rho and Rac [8,10,18,19].

Studies of the other Dact paralogs have yielded similarly conflicting data. Morpholino-based knock-down of Dact2 during zebrafish development produced foreshortened, laterally expanded embryos consistent with a role in the Planar Cell Polarity (PCP) pathway [16]. However, a separate zebrafish study found that Dact2 primarily regulates Activin/Nodal-type TGFβ signaling via binding to the Alk4/5 class of transmembrane receptors, promoting their lysosomal degradation [12]. This conclusion has been supported by subsequent knock-down and gene-targeted deletion of *Dact2 *in mammalian cell lines and mice, which led to modest increases in TGFβ-signaling read-outs and concordant tissue phenotypes [20-22], although some of these phenotypes might also be consistent with disruptions in the PCP pathway. Dact3 was the last paralog to be identified. No reports of its embryonic function have been published but one study showed that the human protein acts as a tumor suppressor in adenocarcinoma cells by repressing Wnt/β-catenin signaling [23].

Given the diverse signaling roles and binding partners ascribed to Dact proteins, a reasonable hypothesis is that distinct protein-protein interactions confer distinct signaling activities onto each Dact paralog. To address this hypothesis, we undertook a systematic study of Dact complex formation in a representative experimental system. We recombinantly expressed identically epitope-tagged versions of each of the three murine and selected non-murine Dact homologs, along with alternately tagged versions of putative interacting proteins in immortalized human embryonic kidney (HEK293 and HEK293T) cell lines. We then conducted co-immunoprecipitation (coIP) assays on cell lysates to analyze protein complex formation in these cells. This assay was chosen because it has been employed previously by several independent groups to verify many of the proposed Dact partners [1,2,8,9,11,12]. CoIPs for each putative interactor were performed under identical conditions in parallel and replicated multiple times. Our chief aim was to characterize conserved protein interactions across paralogous members of the Dact protein family with the hope that this would clarify previously reported findings for individual family members, suggest whether members of this protein family are likely to subserve physiologically conserved or divergent functions, and finally to suggest which signaling or cell biological pathway(s) is most likely to be involved.

## Results and Discussion

### Dacts are phosphoproteins that migrate at higher than expected molecular weight on SDS-PAGE

**Figure 1:**
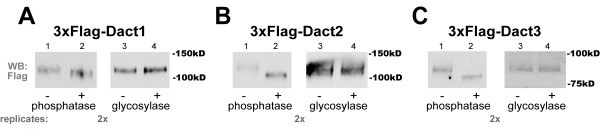
**All Dacts run at higher than expected molecular weight and are phosphorylated in vivo**. Lysates from HEK293 cells transiently transfected with plasmids expressing N-terminal FLAG-tagged murine Dact proteins, enzymatically treated as indicated, separated by SDS-PAGE, and visualized by immunoblotting with anti-FLAG antibody. All panels: lanes 1 & 3 untreated, lane 2 treated with phosphatase, Lane 4 treated with glycosylase. **A**, Dact1. **B**, Dact2. **C**, Dact3. In this and all subsequent figures, the number of replicate experiments is indicated in grey beneath each corresponding panel.

Some previous studies and commercial antibody sources have reported apparent molecular weights for full-length Dact1 proteins as less than 100 kD [2,9] consistent with bioinformatic predictions based on primary sequence information but inconsistent with our previously published biochemical data [1,8]. Using SDS-PAGE, recombinantly expressed full-length Dact1 and Dact2 consistently migrate between 100-120 kD [1,8] and Dact3 migrates between 75-100 kD [4,23]. Part of the apparent discrepancy between bioinformatic prediction and experimental observation is due to phosphorylation in vivo [1,11], as demonstrated by a downward mobility shift when cell lysates containing Dact proteins are pan-dephosphorylated (Figure 1A-C, left panels). Since even pan-dephosphorylated Dact proteins migrate at a larger than expected size, we checked for evidence of other post-translational modifications that can variably affect apparent molecular weight by SDS-PAGE, such as glycosylation. However, treatment of Dact paralogs with an enzymatic deglycosylation cocktail caused no shift in their apparent molecular weight (Figure 1A-C, right panels), nor could we detect any evidence of glycosylation using dye-based methods such as periodic acid-Schiff staining (data not shown).

### All murine Dact paralogs form complexes with CK1δ/ε homologs

**Figure 2:**
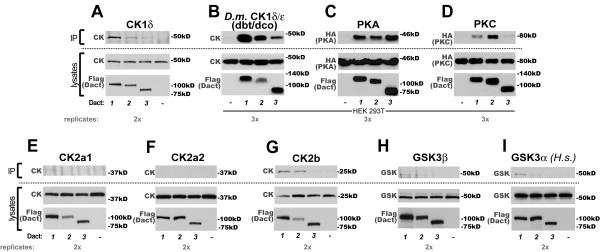
**All Dacts form robust complexes with CK1δ/ε, PKA and PKC; less so with other serine/threonine kinases**. In this and all subsequent figures, lysates from cells transiently transfected with plasmids expressing N-terminal FLAG-tagged murine Dact proteins or no Dact (negative control) plus the indicated interactor, underwent coIP using anti-FLAG antibody, were separated by SDS-PAGE, and proteins visualized by immunoblotting with the indicated antibody. All cells are HEK293 unless indicated as HEK293T. Unless otherwise indicated all proteins are murine (or identical to murine). Top = coIP immunoblot, bottom = lysate immunoblots (confirming appropriate protein expression), lanes as marked: 1 = Dact1, 2 = Dact2, 3 = Dact3,-= negative control. **A**, CK1δ. **B**, *D. melanogaster *CK1δ/ε (dbt/dco). **C**, PKA. **D**, PKCγ. **E**, CK2a1. **F**, CK2a2. **G**, CK2b. **H**, GSK3β. **I**, GSK3α (human).

One of the initial reports identifying Dact1 in *Xenopus laevis *documented complex formation with CK1ε when the protein was expressed in mammalian cell lines [1]; a later study showed that CK1δ-mediated phosphorylation of the *X. laevis *Dact1 protein alters its Wnt/β-catenin signaling activity in a cell-free system [17]. We tested whether interaction with CK1δ/ε was specific to Dact1 or a general feature of all Dact family members. When recombinantly expressed in HEK293 cells, all three murine Dact paralogs formed complexes with murine CK1δ (Figure 2A). We reasoned that if this interaction were functionally important it might occur with more divergent members of the CK1δ/ε family, such as the single CK1δ/ε homolog doubletime/discs overgrown (dbt/dco) from *Drosophila melanogaster*, in which no Dact homolog has yet been identified. Indeed, all three murine Dact paralogs formed robust complexes with *Drosophila *dbt/dco (Figure 2B).

Similarly, Protein Kinase A (PKA) has recently been reported to associate with human DACT1 in HEK293T cells, regulating its activity in Wnt/β-catenin signaling [11]. Concordantly, we found that the catalytic subunit of Protein Kinase A formed complexes with all three murine Dact family members when co-expressed in HEK293T cells (Figure 2C). Protein Kinase C (PKC) has not previously been tested for interactions with Dact proteins, but has been implicated repeatedly in different types of Wnt signaling [24-29]. We found that it formed complexes with all three Dact paralogs when expressed in HEK293T cells-most robustly with Dact2, followed by Dact1 (Figure 2D).

Of the serine/threonine kinases tested, the most robust and conserved interactions were with CK1δ/ε, PKA, and PKC. In contrast, Casein Kinase 2a1 (CK2a1) formed a weak complex only with Dact1 (Figure 2E). Casein Kinase 2a2 (CK2a2) showed no appreciable complex formation with any murine Dact family member (Figure 2E, F). Casein Kinase 2b (CK2b) formed complexes only with Dact1 and Dact2 (Figure 2G). GSK3β, which is central to Wnt/β-catenin signaling and has been reported to interact with Dact1 [1,2], in our assays formed complexes only weakly with Dact1 and not appreciably with either Dact2 or Dact3 (Figure 2H). GSKα behaved indistinguishably from GSKβ in this respect (Figure 2I).

### All murine Dact paralogs form complexes with all Dvl homologs

**Figure 3:**
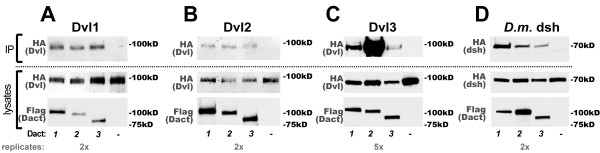
**All Dacts form robust complexes with all Dvls**. **A**, Dvl1. **B**, Dvl2. **C**, Dvl3. **D**, *D. melanogaster *dsh.

Though homologous in the sequences and positions of a few well-conserved domains, the three mammalian Dact paralogs are nevertheless only modestly conserved across their overall primary sequence (~20% identity), and have distinct though overlapping domains of tissue expression during development and in the adult [4]. In contrast, the three mammalian Dvl paralogs are more conserved at the primary sequence level (> 60% identity) and are ubiquitously or near-ubiquitously expressed during development and in adult tissues [30-32]. This, combined with evidence that different Dact paralogs have distinct signaling functions in vivo [16,22], raises the question of whether some Dact paralogs might preferentially associate with only a subset of co-expressed Dvl proteins, or perhaps not associate with Dvl proteins at all. We tested this hypothesis and found that all three murine Dact paralogs formed complexes with all three murine Dvl paralogs (Figure 3A-C). Furthermore each Dact paralog formed complexes with each Dvl paralog indiscriminately, with the sole exception that Dact2 reproducibly showed a particularly strong interaction with Dvl3 (Figure 3C, lane 2 *vs*. lanes 1 & 3). As with CK1δ/ε, all three Dact paralogs also formed complexes with the *D. melanogaster *Dvl homolog, dsh (Figure 3D).

### All Dact paralogs form complexes with Vangl proteins; TGFβ receptor interaction is relatively weaker

**Figure 4:**
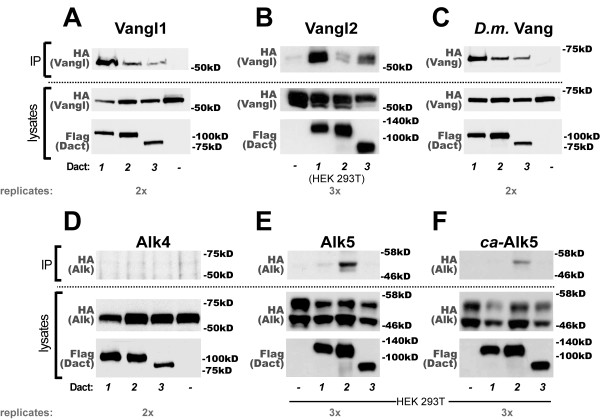
**All Dacts form robust complexes with Vangl family members; less so with TGFβ receptors**. **A**, Vangl1. **B**, Vangl2. **C**, *D. melanogaster *Vang/Stbm. **D**, Alk4. **E**, Alk5. **F**, point-mutated (constitutively active) Alk5.

In the mouse embryo, constitutive loss of Dact1 leads to post-translational upregulation of the Vangl2 transmembrane protein in cells undergoing epithelial-to-mesenchymal transition at the primitive streak with consequences on gastrulation and subsequent morphogenic events in the posterior mesoderm and endoderm [8]. This finding in genetically-engineered mice led to our discovery that in addition to the Dvl proteins that bind to Vangl2 [33,34], Dact1 binds to Vangl2 via independent domain interactions [8]. There are two paralogous Vangl proteins in mammals (Vangl1 and Vangl2) that at least partially overlap in function [35]. We accordingly tested the hypothesis that all Dact paralogs can form complexes with Vangl paralogs. We found that all three Dact proteins formed robust complexes with Vangl1 (Figure 4A). However, to our surprise there were some differences in the affinity of each murine Dact protein for Vangl2. Specifically, by coIP assay Dact1 formed the most robust complexes with Vangl2 (both in HEK cells, not shown; and in HEK293T cells, shown), followed by Dact3, and then by Dact2 which formed complexes with Vangl2 at levels just detectable above background (Figure 4B). As with the CK1δ/ε and Dvl protein families, all three murine Dact paralogs readily formed complexes with the sole *D. melanogaster *Vangl family member, Vang/Stbm (Figure 4C).

Dact2 has been implicated in TGFβ signaling via binding, endocytosis, and lysosomal degradation of the Alk4/5 subtype of TGFβ receptor proteins [22]. Combined with the observations above regarding Dact protein binding to the Vangl transmembrane protein family, this raises the possibility that Dact proteins might be involved in endocytic turnover and degradation of multiple classes of transmembrane protein. We therefore sought to replicate complex formation between Dact2 and Alk5, and also asked whether all Dact proteins interact similarly with TGFβ receptors. Relative to the Vangl proteins, we observed weaker complex formation between murine Dact proteins and Alk5. In HEK293 cells we were unable to detect complex formation between Alk4 or Alk5 and any Dact protein (Figure 4D and data not shown). In HEK293T cells we could replicate weak complex formation between both the wild type and a constitutively active point-mutated form of Alk5 [36] (Figure 4E, F); the coIP of Alk5 was weakly positive with Dact1, and negative with Dact3 (Figure 4D-F).

### Complex formation with catenin proteins is relatively weak and most conserved for p120ctn

**Figure 5:**
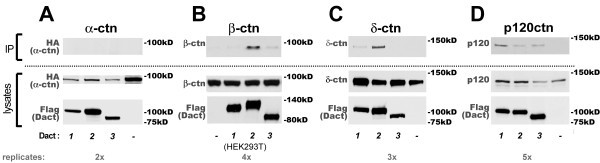
**Dacts weakly coIPs with some catenin family members**. **A**, α-catenin. **B**, β-catenin. **C**, δ-catenin. **D**, p120-catenin.

When co-expressed in tissue culture cells Dact1 can form complexes with β-catenin [1,2] and this interaction has been mapped to the β-catenin armadillo repeat region [2], a structurally-conserved protein-interaction domain shared with other members of the catenin superfamily as well as with other proteins [37]. Dact1 has also been shown to bind and regulate the catenin p120ctn [10]. We therefore tested interactions between the three murine Dact paralogs and representatives from each major class of the catenin superfamily. No Dact paralogs formed complexes with α-catenin (Figure 5A), which lacks armadillo repeats. In contrast, Dact2 and Dact3 formed complexes, albeit weakly, with β-catenin in HEK293T cells; Dact2 exhibited the stronger β-catenin coIP (Figure 5B). Dact2 also showed the strongest coIP with δ-catenin; Dact1 interacted weakly whereas complex formation between δ-catenin and Dact3 was not detectable above background (Figure 5C). Among members of the catenin superfamily, the Dact interaction that was most conserved was with p120ctn (Figure 5D). Notably, even positive coIPs with catenin superfamily members were less robust than those with CK1δ/ε, Dvl, or Vangl family members.

### A subset of Dact proteins weakly complexes with LEF/TCF proteins and with HDAC1

**Figure 6:**
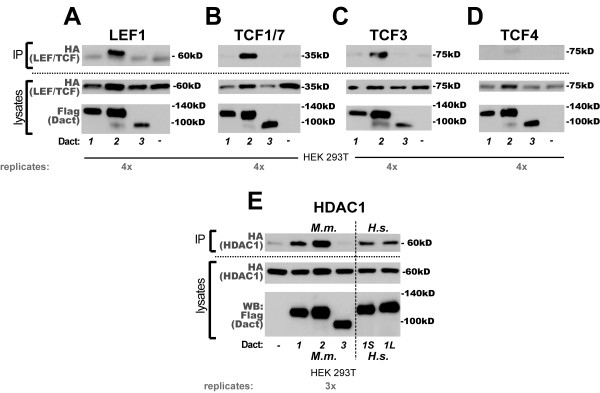
**Dact2 coIPs with LEF/TCF family members; Dact1 & 2 coIP with HDAC1**. **A**, LEF1. **B**, TCF1/TCF7. **C**, TCF3. **D**, TCF4. **E**, HDAC1: left 4 lanes murine, right two lanes human: 1S = human Dact1 short isoform, 1L = human Dact1 long form.

The Dact1 homologs from *X. laevis *and *H. sapiens *have been reported to form complexes with a subset of the LEF/TCF transcription factors that act as transcriptional regulators downstream of Wnt/β-catenin signaling and some other pathways [2,7,10]. We sought to replicate this finding and to test its specificity for Dact1 versus the other two Dact paralogs. Using the 293T cell line, we detected a positive coIP only for murine Dact2; this interaction was positive across all members of the LEF/TCF family examined (Figure 6A-D).

Another nuclear protein that has been reported to interact with DACT1 from *H. sapiens *is HDAC1 [2]. Using the HEK293T cell line and the murine Dact paralogs, we could replicate this finding for Dact1, but found that the coIP was stronger between Dact2 and HDAC1, whereas with Dact3 it was not detectable above background (Figure 6E, left). Because the previously published experiment was performed with human homologs in HEK293T cells, we replicated this for both the short and long isoforms of human DACT1 (Figure 6E, right).

### All Dact proteins homo- and hetero-dimerize

Given numerous efforts by several independent groups to experimentally identify novel Dact interacting proteins, it is curious that no binding partner for one of the principal conserved Dact domains has been identified, specifically the leucine zipper region near the N-terminus. The leucine zipper is a well-defined structural motif that forms an amphipathic alpha helix or coiled-coil with a hydrophobic stripe along one side; this acts as a protein interaction or dimerization domain [38,39]. Given the established ability of leucine zippers to mediate dimerization and the lack of a putative partner for this domain in Dact family members, we hypothesized that this conserved domain might mediate Dact homo- and/or hetero-dimer formation.

**Figure 7:**
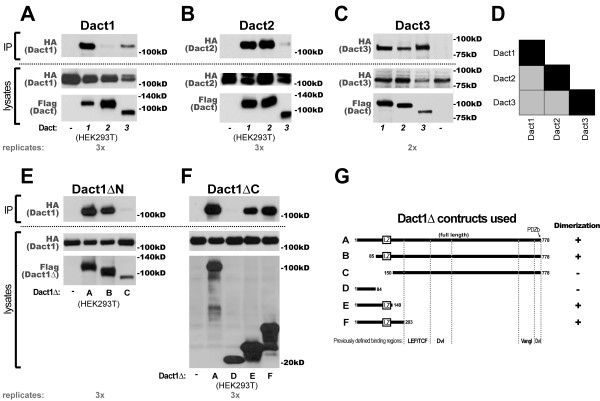
**All Dacts homo- and hetero-dimerize mediated by their leucine zipper domains**. **A**, Dact1. **B**, Dact2. **C**, Dact3. **D**, Schematic summary of interactions: shading indicates relative strength of coIP; homo-interaction coIPs are modestly more robust than hetero-interaction coIPs. **E-G **The leucine zipper domain is necessary and sufficient for Dact1 homo-interactions: **E**, Dependency on the Dact1 N-terminus analyzed using serial N-terminal truncations. **F**, Dependency on the Dact1 C-terminus analyzed using serial C-terminal truncations. **G**, Schematic summary of results from **E & F**.

We tested this hypothesis using the same experimental strategy used above to assess other potential interactions: we co-expressed alternately tagged murine Dact paralogs in HEK293 or 293T cells and performed coIPs, pulling down complexes with one epitope tag and probing gel-separated precipitated protein complexes with the other. We found that all Dact paralogs form complexes with themselves and with other Dact paralogs (Figure 7A-C). In general coIPs involving Dact homo-interactions were moderately more strongly positive than hetero-interactions (Figure 7A-C, summarized in Figure 7D). Using two panels of Dact1 deletion constructs, one incorporating successive deletions at the N-terminus (Figure 7E) and the other incorporating successive deletions at the C-terminus (Figure 7F) we confirmed that the leucine zipper region of Dact1 is both necessary and sufficient for this association, consistent with leucine-zipper mediated dimerization (summarized in Figure 7G).

## Conclusions

### Overview

Our data indicate that the most robust interactions for all mouse Dact paralogs are with members of the Dvl and Vangl protein families; these interactions, along with interactions with several kinases, are conserved across all members of the Dact gene family. Somewhat surprisingly, the Dvl, Vangl, and Casein Kinase 1δ/ε (CK1δ/ε) proteins derived from the fruit fly *Drosophila melanogaster*, in which a Dact paralog has yet to be identified, also readily formed complexes with mammalian Dact paralogs. We also discovered that all Dact proteins can form complexes with themselves and with each other, and their conserved leucine zipper domains are necessary and sufficient for this interaction, suggesting dimerization. This has implications for functional cooperation between Dact family members, particularly in those tissues where the paralogs are co-expressed. It also raises the possibility that mutant or overexpressed Dact proteins could cause dominant effects by association and interference with wild type Dact proteins and their partners. Taken together, our biochemical findings suggest that all Dact family members participate in conserved kinase-regulated biochemistry involving Vangl and Dvl. This suggests a role within, or upstream of, PCP or a molecularly related pathway. It further suggests that some mutations in the human *DACT *loci could contribute to pathogenesis by disrupting this conserved pathway in a dominant or semi-dominant manner.

### Functional Implications of Dact Phosphorylation

We suspect that the smaller sizes reported for Dact1 homologs in some studies and commercial antibody literature may variously represent poorly resolved size-markers, partial proteolysis products, and/or non-specific antibody cross-reactivity to more abundant cellular proteins. Dact proteins all clearly interact with several kinases, including not only CK1δ/ε and PKA, but also PKC and possibly other kinases as well. Phosphorylation and other post-translational modifications of Dact proteins may regulate function [11,17]; this idea is certainly worthy of further empirical exploration not restricted to Wnt/β-catenin signaling, as that may not be the sole or even the primary physiological role for this protein family. For example, we and others have not yet tested whether Dact proteins can interact with or are modified by tyrosine kinases, some of which have recently been shown to play important roles in PCP signaling [40]. We note that at least one highly conserved peptide motif in the Dact proteins, located just C-terminal to the leucine zipper domain, contains not only two serines but also an invariant tyrosine in all family members [4].

### Conserved binding partners suggest conserved function in a conserved pathway

Conservation of the most robust coIP partners among the Dact paralogs (Table 1) suggests that this protein family plays a conserved role in kinase-regulated cellular biochemistry involving Vangl and Dvl. One candidate pathway consistent with functional data derived from knock-out mice and other model systems is PCP signaling, which regulates cell polarity, adhesion, and migration in many tissues [8,16,18,19,21,24,25,41,42]. If Dact proteins do play such a "core" role in PCP signaling in vertebrates, it is curious that no Dact homolog has yet been discovered in *D. melanogaster *where PCP was first described and where many other core PCP components have been discovered and initially characterized. However, given the limited overall sequence conservation between mammalian Dact paralogs, it is possible that a more divergent Dact family member awaits discovery in the fruit fly. Alternatively, a structurally unrelated protein may play a functionally analogous role to Dact proteins in the PCP pathway in Drosophila. This is supported by our observation that all the murine Dact homologs interact with the unique *D. melanogaster *CK1δ/ε, Dvl, and Vangl homologs.

**Table 1:** Complex formation with Dact1, Dact2, and Dact3

putative interactors (mouse unless other specified)				
**bold = positive for all paralogs**		**Dact1**	**Dact2**	**Dact3**

**Dact family (dimers)**	**Dact1**	**+**	**+**	**+**
	**Dact2**	**+**	**+**	**+**
	**Dact3**	**+**	**+**	**+**

**Dvl family**	**Dvl1**	**+**	**+**	**+**
	**Dvl2**	**+**	**+**	**+**
	**Dvl3**	**+**	**+**	**+**
	**dsh **(fruit fly)	**+**	**+**	**+**

**Vangl** **family**	**Vangl1**	**+**	**+**	**+**
	**Vangl2**	**+**	**-/+**	**+**
	**Vang/Stbm **(fruit fly)	**+**	**+**	**+**

**Casein Kinase 1δ**/ε	**CK1δ**	**+**	**+**	**+**
	**dbt/dco **(fruit fly)	**+**	**+**	**+**

**Protein Kinase A**	**PKA**	**+**	**+**	**+**

**Protein Kinase C**	**PKC**	**+**	**+**	**-/+**

**p120-catenin**	**p120-catenin**	**+**	**+**	**+**

other catenins	α-catenin	**-**	**-**	**-**
	β-catenin	**-**	**+**	**-/+**
	δ-catenin	**-/+**	**+**	**-**

Casein Kinase 2s	CK2a1	**-/+**	**-**	**-**
	CK2a2	**-**	**-**	**-**
	CK2b	**+**	**+**	**-**

GSK3 family	GSK3α (human)	**-/+**	**-/+**	**-**
	GSK3β	**-/+**	**-/+**	**-**

HDAC1	HDAC1	**+**	**+**	**-**

LEF/TCF family	LEF1	**-**	**+**	**-**
	TCF1/TCF7	**-**	**+**	**-**
	TCF3	**-**	**+**	**-**
	TCF4	**-**	**-/+**	**-**

TGFβR family	Alk4	**-**	**-**	**-**
	Alk5	**-/+**	**+**	**-**

It is also possible that the pathway involving Dact proteins in vertebrates is not synonymous with the PCP pathway in Drosophila. A divergent signaling pathway might regulate a catenin protein, such as p120ctn which was positive in our coIP assay with every Dact paralog (Table 1). The p120ctn protein plays a role at the plasma membrane in cytoskeletal and adhesive events [43], at the nucleus in gene transcription [10], and has recently been shown to interact with CK1ε and the Wnt receptor complex in Wnt/β-catenin signaling [44]. Given all this, a transient interaction with Dact proteins reflected by a comparatively weak coIP, but that regulates p120ctn localization or stability, could account for at least some conflicting observations of Dact function derived from different model systems. Alternatively, a more robust and specific functional interaction might exist between Dact proteins and an unidentified armadillo repeat containing protein, of which there are an abundance of candidates both within and without the catenin superfamily [37].

Based on the robustness of interactions between Dact, Dvl, and Vangl proteins, in those cells where these proteins are coexpressed they might be expected to form a stable or semi-stable complex. A logical future direction is to determine the subcellular localization of this putative complex and to identify other colocalized proteins. This will provide clues about whether Dact family members play a primary role in intercellular signaling, extracellular adhesion, cytoskeletal polarity, or perhaps in the protein trafficking that underlies one or more of these cell biological processes [8,9,12,18,45-47]. Indeed, given interactions documented here and elsewhere between Dact proteins and two widely divergent types of transmembrane protein [8,22], as well as evidence that Dvl proteins play a role in endocytic regulation of transmembrane receptors [46,48], a role for Dact proteins in transmembrane protein trafficking merits further investigation. The relatively stronger coIPs of Dact2 with Dvl3 and Alk5, and of Dact1 with Vangl2, support prior suggestions that there is some functional divergence between Dact paralogs [16,22], but this should also be reconsidered in light of the new biochemical evidence presented here that Dact paralogs can physically interact. This suggests that Dact paralogs may functionally cooperate or compete in those cells where they are coexpressed.

### Implications of Dact Dimer Formation

The discovery reported here that Dact paralogs can heterodimerize has implications for their physiological function. Although the mammalian Dact proteins do display distinct patterns of expression, there are many developing and mature tissues in which two or all three paralogs are co-expressed [4]. To the extent that coexpressed Dact proteins form active heterodimers they must functionally cooperate in these tissues. Despite some limited differences, our side-by-side comparison found conserved coIP interactions between every Dact paralog tested and the most robust partnering proteins. Taken together, the conserved coIP profiles and dimerization data suggest that Dact paralogs are likely to participate in shared biochemistry and have convergent physiological functions. If Dact paralogs do differ in endogenous activity, then in those cells where they are coexpressed they might mediate signaling pathway cross-talk and/or antagonism-either through non-productive heterodimer formation or through competition for common binding partners.

The discovery that Dact proteins dimerize also raises important issues for biochemical data interpretation. Immunoblotting and immunohistological data suggest that levels of endogenous Dact proteins are low even in those tissues where the mRNA is present and where knock-down or knock-out causes phenotypes [8,18-21,23]. In healthy tissues the levels of Dact proteins may be tightly regulated because, as self-associating scaffold proteins, if their levels are elevated they can aggregate with themselves, their partners, and with other more loosely associated proteins. In that case, non-physiological effects on biochemical pathways, including Wnt/β-catenin signaling, may occur in heterologous and in vitro assays in which these proteins are not maintained in their native cell biological context or concentrations. Indeed, functional studies in genetically engineered mice [8,19,20] so far do not support previous findings that Dact proteins play roles in Wnt/β-catenin signaling. Nevertheless, it remains possible that the lack of observed impacts on Wnt/β-catenin signaling in single-hit *Dact *mutant mice is due to redundancy between paralogs with respect to this pathway. This will be resolved once phenotypic and signal pathway consequences can be assessed in a mouse line in which all three Dact genes have been simultaneously eliminated. All that said, if Dact proteins are ultimately determined not to physiologically modulate Wnt/β-catenin signaling in healthy tissues, it will remain possible that they do influence this signaling pathway in cancerous and other diseased tissues where their levels or subcellular localization are dysregulated via mutation or epigenetic mechanisms [2,15,23,49,50].

Clinically, the discovery that their translation products homo- and hetero-dimerize raises the possibility that missense mutations in any of the three human *DACT *genes could cause genetically dominant or semi-dominant effects by interfering with functions of wild type homologs produced from unaffected alleles in the same individual. Given evidence that these proteins participate in a conserved biochemical pathway with demonstrated critical roles in urinary and lower gastrointestinal system development [8,21], in neural differentiation and synaptogenesis [19], and in oncogenesis and metastasis [15,23,49], human genetic variants at these loci may have important clinical ramifications.

## Methods

### Cell culture, Transfections, and CoIPs

Performed as described [1,51] with the following modifications. Two different protocols were employed depending on desired stringency. In cases where candidate interactors were not found to detectably coIP with Dact proteins in HEK293 cells (ATCC product number CRL-1573), the experiment was repeated in HEK293T/17 cells (ATCC product number CRL-11268); in some cases only the HEK293T/17 cell line and associated protocol was attempted. Where employed, the HEK293T/17 cell line and coIP protocol is specified in the text and figures as "HEK293T". In brief:

#### HEK293 (Lower plasmid copy number than HEK293T/17 cells, detergent in washes)

Cells were maintained in DMEM supplemented with 10% FCS, 100 units ml^-1 ^penicillin G and streptomycin, and transfected on 10 cm plastic plates (Corning) with Effectene (Qiagen, catalog # 301427) at 80% confluency. Cells were lysed 24 hours post-transfection in lysis buffer (25 mM Tris pH = 8.0, 150 mM NaCl, 1%Triton, 0.2% deoxycholate, 2 mM EDTA) supplemented with protease and phosphatase inhibitors (Sigma-Aldrich, catalog # P8340, P0044+P5726). Supernatant was separated from insoluble material by centrifugation (10 minutes, 14,000 rpm, 4°C), and 3-5% of the total volume set aside for lysate immunoblotting. The remainder was used for coIP: 2 ug of anti-FLAG antibody was added to the supernatant and nutated overnight at 4°C. Protein A/G agarose beads (Santa Cruz Biotechnology, catalog # sc-2003) were then added and nutated for 30 minutes at 4°C to capture immune complexes. Beads were collected by centrifugation (30 seconds, 6000 g) and washed 3 times for 5 minutes each in ice-cold lysis buffer. Washed CoIP protein complexes were eluted in Laemmli protein gel loading buffer and boiled for 5 minutes before separation by Sodium Dodecyl Sulfate Polyacrylamide Gel Electrophoresis (SDS-PAGE).

#### HEK293T (Higher plasmid/protein expression levels than HEK293 cells, no detergent in washes)

Cells were maintained as above, but plated at a density of 1 × 10^6 ^cells in 60 mm culture dishes and allowed to grow for 12 hours before transfection using Lipofectamine 2000 (Invitrogen, catalog # 11668-019). Cells were harvested and lysed 48 hours post-transfection in a buffer containing 50 mM Tris-HCl, pH 7.4, 150 mM NaCl, 1 mM EDTA, and 1%Triton X-100 supplemented with EDTA-free protease inhibitor tablets (Roche, catalog # 11836170001). Supernatant and lysate sample were prepared as above. Supernatant was pre-cleared by incubating with mouse IgG-agarose bead (Sigma-Aldrich, catalog # A0919) for 1 hour at 4°C with tumbling. Cleared lysate was then mixed with anti-FLAG M2-conjugated agarose beads (Sigma-Aldrich, catalog # A2220) and rotated in an Eppendorf tube at 4°C for 3 hours. Beads were collected as above but washed 3 times for 10 minutes each in ice-cold TBS (50 mM Tris HCl, 150 mM NaCl, pH 7.4). Washed protein complexes were eluted and separated by SDS-PAGE as above.

### Phosphatase Treatment

Whole cell extracts from transfected cells in lysis buffer without phosphatase inhibitors were treated with lambda protein phosphatase (New England Biolabs, catalog # P0753) for 30 minutes at 30°C. Reactions were blocked by boiling in Laemmli protein gel loading buffer and resolved by SDS-PAGE.

### Deglycosylation

Whole cell extracts from transfected cells in lysis buffer were treated with a protein deglycosylation mix (New England Biolabs, catalog # P6039) according to manufacturer's instructions. Reactions were blocked by boiling in Laemmli protein gel loading buffer and resolved by SDS-PAGE.

### cDNAs and Expression Plasmids

The three murine Dact cDNAs employed in this study have been described previously [4]. The human short DACT1 isoform (GenBank NM_001079520.1) was obtained by RT-PCR from HEK293T cells, and the long DACT1 isoform (GenBank NM_016651.5) was synthesized from the shorter clone using overlapping PCR. The human GSK3α cDNA was obtained from Dr. Junichi Sadoshima (UMDNJ). All other cDNAs were obtained commercially from Open Biosystems (*M. musculus *clones), from the Bloomington Stock Center (*D. melanogaster *clones), or were generated in the Cheyette laboratory by RT-PCR from total mouse embryonic mRNA. For transfection and expression in cells, all Dact cDNAs were subcloned into vector p3XFLAG-CMV-10 (Sigma-Aldrich, catalog # E7658) whereas all putative interactor cDNAs were subcloned into vector pcDNA3.1(-) (Invitrogen, catalog # V795-20). The sequence of each cDNA employed was confirmed by Sanger sequencing.

### Antibodies

The provenance of all commercial antibodies employed in this study is shown in Table 2. Immunoblots were generally incubated with primary antibodies overnight at 4°C in 5% milk in TBST.

**Table 2:** Provenance of antibodies used

Epitope	Company	Catalog #
FLAG	Sigma-Aldrich	F1804

HA	Roche	11867423001

CK1δ	Santa Cruz Biotechnology	SC6473

CK2a1	Santa Cruz Biotechnology	SC6479

CK2a2	Santa Cruz Biotechnology	SC6481

CK2b	Santa Cruz Biotechnology	SC46666

GSK3α/β	Santa Cruz Biotechnology	SC7291

β-catenin	BD Biosciences	610153

δ-catenin	BD Biosciences	611536

p120-catenin	BD Biosciences	610133

## List of abbreviations

CK: Casein Kinase; coIP: co-immunoprecipitation; Dact: Dapper antagonist of catenin; dbt/dco: doubletime/discs overgrown; Dvl: Dishevelled; GSK: Glycogen Synthase Kinase; HDAC1: Histone Deacetylase 1; HEK: Human Embryonic Kidney; LEF/TCF: Lymphoid Enhancing Factor/T Cell Factor; p120ctn: p120-catenin; PCP: Planar Cell Polarity; PDZ: Postsynaptic density-95/Discs large/Zonula occludens-1; PKA: Protein Kinase A; PKC: Protein Kinase C; SDS-PAGE: Sodium Dodecyl Sulfate Polyacrylamide Gel Electrophoresis; TGFβ: Transforming Growth Factor β; Vang/Stbm: Van Gogh/Strabismus; Vangl: Van Gogh-like.

## Authors' contributions

SK, XYY, and BNRC conceived the experiments. SK and XYY performed all the experiments, including construction of any novel reagents or analysis tools. BNRC wrote the manuscript, which all the authors reviewed, edited, and approved.
